# Quality of Life in Patients with Treatment-Resistant Depression Treated with Intranasal Esketamine as Add-On Therapy to an SSRI—A Prospective, Comparative Study in Real-World Clinical Practice

**DOI:** 10.3390/ph19101644

**Published:** 2026-10-19

**Authors:** Daniel Szawarnoga, Joanna Fojcik, Michał Górski, Marek Krzystanek

**Affiliations:** 1 Department of Psychiatry, Department of Neurology, Faculty of Health Sciences in Katowice, Medical University of Silesia in Katowice, 40-752 Katowice, Poland; joanna.fojcik@sum.edu.pl (J.F.); michal.gorski@sum.edu.pl (M.G.); 2 Department of Clinical and Community Psychiatry and Psychology, Collegium Medicum, WSB University, 41-300 Dąbrowa Górnicza, Poland; krzystanekmarek@gmail.com

**Keywords:** treatment-resistant depression, esketamine, quality of life, WHOQOL-BREF, SF-36, real-world evidence, patient-reported outcomes, antidepressant treatment

## Abstract

Treatment-resistant depression (TRD) remains one of the most clinically challenging forms of depressive disorders. In many patients, reduction in symptom severity does not translate into full functional recovery; therefore, assessment of treatment effectiveness should also include health-related quality of life (HRQoL). **Objective**: The aim of the study was to compare changes in HRQoL in patients with TRD receiving a treatment strategy including intranasal esketamine as add-on therapy to ongoing treatment based on selective serotonin reuptake inhibitors (SSRIs) and in patients treated with SSRI-based therapy without esketamine. **Materials and Methods**: A prospective, observational, comparative cohort study was conducted in real-world clinical practice. Sixty adult patients were included: 30 received intranasal esketamine as add-on therapy to ongoing SSRI-based treatment and 30 received SSRI-based treatment without esketamine. The study protocol did not impose a separate SSRI dose-optimization algorithm; drug selection and dose modifications remained at the discretion of the treating physician in accordance with routine clinical practice. Quality of life was assessed using the World Health Organization Quality of Life-BREF (WHOQOL-BREF) and the 36-Item Short Form Health Survey (SF-36). Depressive symptom severity was assessed using the Montgomery–Åsberg Depression Rating Scale (MADRS) and the Hamilton Depression Rating Scale (HDRS). Assessments were performed at baseline, weeks 4, 8, and 12, and after 12 months. **Results**: Both groups showed improvement in quality of life and reduction in depressive symptom severity, with larger absolute changes in the group receiving esketamine. Differences involved WHOQOL-BREF domains and selected standard SF-36 subscales. **Conclusions**: In this observational cohort, the treatment strategy including intranasal esketamine was associated with greater improvement in several HRQoL measures than SSRI-based treatment without esketamine. Because treatment exposure was not assigned by the investigators and residual confounding cannot be excluded, the findings should not be interpreted as evidence of an isolated causal effect of esketamine.

## 1. Introduction

Depression is a major clinical and public health problem. Its significance stems not only from the high prevalence and presence of depressive symptoms, but primarily from their impact on the patient’s functioning: work capacity, social activity, family rela-tionships, sleep, energy, motivation, and overall sense of agency. In clinical practice, patients do not simply expect a decrease in their depression score; they expect a return to a life they find acceptable [1].

Treatment-resistant depression (TRD) is most commonly defined as failure to re-spond to at least two adequate antidepressant treatment trials administered at appro-priate doses for an appropriate duration. This population is particularly burdened clin-ically: patients more frequently experience relapses, longer episodes, comorbid anxiety and somatic symptoms, reduced occupational activity, and increased social and healthcare-system costs [2,3,4,5]. Treatment resistance remains an incompletely under-stood clinical phenomenon. One contributing factor may be insufficient consideration of the impact of depressive symptoms on patient functioning, which may contribute to treatment discontinuation and nonadherence.

Selective serotonin reuptake inhibitors (SSRIs) remain one of the primary antide-pressant classes and are considered first-line medications in the treatment of depression. Their importance stems from their availability, clinical experience, and relatively good tolerability profile. However, clinical trial data show that a significant proportion of patients do not achieve response or remission after subsequent treatment phases, and the symptomatic improvement achieved may not fully translate into improved quality of life [6].

Intranasal esketamine (ESK), the S-enantiomer of ketamine and an antagonist of the N-methyl-D-aspartate (NMDA) receptor, introduced a distinct mechanism of action into the treatment of treatment-resistant depression. The TRANSFORM and SUSTAIN studies demonstrated the efficacy of esketamine used together with an oral antidepres-sant, while the ESCAPE-TRD study provided important comparative data versus aug-mentation with extended-release quetiapine (XR) [7,8,9,10,11,12,13,14]. Current Canadian Network for Mood and Anxiety Treatments (CANMAT) guidelines include intranasal esketa-mine as an add-on treatment option for patients with an inadequate response to at least two adequate antidepressant trials [15]. From the perspective of routine clinical prac-tice and the patient, however, it is important not only whether a treatment reduces symptom severity, but also whether the patient begins to function better. Incorporating the subjective dimension of functional improvement, reflected in quality of life, enables a more comprehensive assessment of remission from depression.

Assessment of health-related quality of life (HRQoL) is particularly important in this context. The World Health Organization Quality of Life-BREF (WHOQOL-BREF) describes quality of life across physical, psychological, social, and environmental do-mains, whereas the 36-Item Short Form Health Survey (SF-36) enables a more detailed assessment of health functioning, role limitations, emotional well-being, and social functioning. These instruments complement traditional depression scales by showing whether clinical improvement translates into the patient’s daily life [16,17].

Despite the growing number of randomized controlled trials (RCTs), data from re-al-world clinical practice are still needed. RCTs provide high internal validity but typi-cally involve selected populations. Real-world evidence allows for the assessment of treatment in settings closer to everyday care, involving patients with comorbidities and the real-world organizational constraints of the healthcare system [18,19].

The primary objective of the study was to compare 12-month changes in HRQoL across WHOQOL-BREF domains and standard SF-36 subscales between patients re-ceiving a treatment strategy including intranasal ESK as add-on therapy to SSRI-based treatment and patients treated with SSRI-based therapy without esketamine. The sec-ondary objective was to describe longitudinal changes in depressive symptom severity measured with the MADRS and HDRS and to interpret these changes in relation to the HRQoL trajectory. We hypothesized that the ESK-inclusive strategy would be associ-ated with greater improvement in HRQoL.

## 2. Results

The obtained results were divided into two groups—characteristics of changes in depressive symptoms and changes in the quality of life of the studied patients.

### 2.1. Dynamics of Depressive Symptoms

Reductions in MADRS and HDRS scores were observed in both groups, but the improvement was greater in the ESK group. At endpoint, the mean MADRS score was 10.2 ± 3.8 in the ESK group and 16.5 ± 4.7 in the SSRI group. For the HDRS, the endpoint scores were 8.1 ± 3.2 and 13.7 ± 4.0, respectively. Data are presented in Table 1.

### 2.2. Descriptive WHOQOL-BREF Summary Indicator

The descriptive WHOQOL-BREF summary indicator showed a larger absolute change in the ESK group than in the SSRI group. The value increased from 52.1 ± 6.3 to 75.4 ± 5.7 in the ESK group (+23.3 points) and from 51.7 ± 6.0 to 67.2 ± 5.9 in the SSRI group (+15.5 points); the between-group difference in absolute change was 7.8 points (nominal *p* = 0.01). This indicator was treated as descriptive only; the primary interpretation of WHOQOL-BREF was based on the individual domains, and the relative point change was not assumed to represent a standardized effect size directly comparable with other quality-of-life instruments. Descriptive results for the WHOQOL-BREF summary score in the ESK and SSRI groups are presented in Table 2.

Neither the Physical Component Summary (PCS), the Mental Component Summary (MCS), nor any other validated overall SF-36 index was calculated. Accordingly, the nonstandard SF-36 “overall index” was removed from inferential interpretation, and SF-36 findings were reported only at the level of the standard subscales.

### 2.3. WHOQOL-BREF Domains

Analysis of the WHOQOL-BREF domains showed multidimensional improvement in both groups, with larger absolute changes in the ESK group. The largest changes in the ESK group were observed for physical health (+25.5 points), social relationships (+23.5 points), and psychological health (+23.4 points). Because four domain-level comparisons were performed, nominal *p* values were additionally adjusted using the Holm procedure to control the family-wise type I error rate. Both nominal and Holm-adjusted *p* values are reported. The direction and statistical significance of the observed domain-level differences were retained after correction. Detailed results are shown in Table 3.

### 2.4. SF-36 Subscales

In the SF-36 analysis, the ESK group showed larger absolute improvements in selected subscales. The largest changes were observed in physical functioning (+28.3 points), role limitations due to physical health (+25.0 points), mental health/emotional well-being (+24.5 points), and social functioning (+24.2 points). Because five subscale-level comparisons were performed, nominal *p* values were additionally adjusted using the Holm procedure to control the family-wise type I error rate; both nominal and adjusted values are reported. Data are presented in Table 4.

### 2.5. Safety and Tolerability

Both treatment strategies were generally well tolerated. In the ESK group, the most frequently documented adverse events were mild-to-moderate headache (6/30; 20%), transient disorientation or dizziness (5/30; 17%), nausea or vomiting (4/30; 13%), somnolence or fatigue (3/30; 10%), anxiety or inner restlessness (2/30; 7%), and transient blood-pressure increase (2/30; 7%). In the SSRI group, headache (3/30; 10%), nausea or vomiting (2/30; 7%), somnolence or fatigue (5/30; 17%), and anxiety or inner restlessness (4/30; 13%) were documented. No serious adverse events were recorded in either group. Planned treatment and the 12-month assessment were completed by 29/30 patients in the ESK group and 28/30 in the SSRI group. Esketamine was administered under medical supervision, with post-dose observation and blood-pressure monitoring. Dissociative symptoms were not assessed using a dedicated standardized scale, and cognitive function was not a prespecified endpoint; therefore, dissociation rates and cognitive changes are not reported as quantitative outcomes, and transient disorientation/dizziness was not considered equivalent to dissociation. Safety data were descriptive and were not the primary study endpoint.

### 2.6. Retention and Missing Data

Sixty participants were included (30 per group). At 12 months, data were available for 29/30 participants in the ESK group (96.7% retention) and 28/30 in the SSRI group (93.3%). Thus, one participant in the ESK group and two in the SSRI group did not contribute data to the final assessment. Primary analyses were based on available observations at the respective time points. Because the amount of follow-up missingness was small, missing outcome values were not imputed in the primary analyses. The potential for attrition bias was considered when interpreting long-term outcomes.

### 2.7. Treatment Exposure During Follow-Up

The dosing schedule described in Section 4.3 represented the treatment framework, while administration frequency after the induction phase was individualized according to clinical response and tolerability. Final 12-month data were available for 29/30 participants in the ESK group and 28/30 in the SSRI group. The cumulative number of administrations, short treatment interruptions, esketamine dose modifications, and longitudinal changes in oral antidepressant dosing/adherence were not prospectively captured as standardized research variables and are therefore not presented as quantitative exposure measures. Retention in follow-up should not be interpreted as uninterrupted receipt of every scheduled dose.

## 3. Discussion

The main finding of this study was that the real-world treatment strategy including intranasal ESK as add-on therapy was associated with larger improvements in several health-related quality-of-life measures than an SSRI-based strategy without esketamine. A more favorable pattern in the ESK group was observed across WHOQOL-BREF domains and selected SF-36 subscales. Reductions in MADRS and HDRS scores indicate that improvement in clinician-rated depressive symptoms was accompanied by improvement in patient-reported functioning and quality of life. Because the study was observational, nonrandomized, and unblinded, the findings should be interpreted as associations between treatment strategy and outcomes rather than evidence of an isolated causal effect of esketamine.

The obtained results are consistent with the few previous studies examining the effect of esketamine on quality of life and functioning in patients with treatment-resistant depression. In an analysis of data from the TRANSFORM-2 study, Jamieson et al. demonstrated that the use of intranasal esketamine in combination with an oral antidepressant was associated with greater improvements in health-related quality of life, general health status, and functioning than the use of an oral antidepressant with a placebo. After 28 days of treatment, beneficial changes included mobility, self-care, performance of usual activities, and severity of pain and discomfort, as well as reduction in depression-related disability assessed by the Sheehan Disability Scale (SDS). Although this study used different instruments than the present study, primarily the EQ-5D-5L and SDS, the direction of the observed changes is consistent with the results obtained here [14].

Direct numerical comparison of changes in WHOQOL-BREF with EQ-5D-5L or SF-12 is not methodologically justified because these instruments differ in construct, scoring range, and psychometric properties, and the cited studies also use different follow-up time points. In particular, a 12-month change observed in the present study should not be interpreted as equivalent to the 28-day change in the EQ-5D-5L utility index reported in TRANSFORM-2. At the same time, the large absolute changes observed in both groups warrant caution. In a nonrandomized and unblinded observational study, regression to the mean, natural fluctuation of the depressive episode, intensity of clinical contact, concomitant treatment changes, patient expectations, and selective retention may all contribute to the observed magnitude. Accordingly, the reported values are not interpreted as an isolated esketamine effect or as changes directly comparable with those obtained using other quality-of-life instruments.

Similar observations come from long-term analyses of the SUSTAIN-2 program. These studies demonstrated that improvements in depressive symptoms were accompanied by improvements in functioning and quality of life, with a significant portion of the beneficial changes occurring during the induction phase and largely maintained during the optimization and maintenance phases. These data are important for interpreting the results of this study, as they suggest that the potential benefits of esketamine may include not only short-term reductions in depressive symptoms but also broader improvements in functioning and quality of life with continued treatment [20].

Of particular importance are the latest data from the ESCAPE-TRD study, which directly assessed the effect of intranasal esketamine on health-related quality of life. Reif et al. demonstrated greater improvement in selected SF-36 domains in patients receiving intranasal esketamine compared with extended-release quetiapine after 32 weeks of treatment. Differences included social functioning, mental health, and role limitations resulting from emotional problems. Importantly, the results obtained approached levels observed in the general population. Furthermore, clinically significant improvements in the depression-specific QLDS quality of life scale were achieved by 60.7% of patients treated with esketamine compared with 41.8% of patients treated with quetiapine XR. A benefit in favor of esketamine was also observed in the EQ-5D-5L, with a higher proportion of patients reporting no problems in all assessed domains [21].

The convergence of the results of this study with those from TRANSFORM-2, SUSTAIN-2, and ESCAPE-TRD suggests that the benefits of esketamine therapy may extend beyond merely reducing the severity of depressive symptoms. This is particularly important in treatment-resistant depression, where treatment efficacy should be considered not only in terms of symptom response or remission but also through the lens of functional recovery and improved quality of life. In this context, patient-reported outcomes (PROs) are an important complement to traditional psychiatric research endpoints, as they allow for the assessment of whether changes in clinical status translate into areas of functioning that are important from the patient’s perspective [14,21].

It is also worth emphasizing the practical dimension of the observed improvement in quality of life. In the TRANSFORM-2 study, beneficial changes included not only global health assessment but also mobility, self-care, and the ability to perform usual activities, while also reducing depression-related disability [20]. This suggests that improved HRQoL may reflect a partial return to daily functioning rather than solely a subjective reduction in symptom severity. A similar pattern emerged from the ESCAPE-TRD study, in which esketamine treatment was associated with a greater number of weeks in functional remission and less loss of productivity compared with quetiapine XR. In an analysis covering 32 weeks of treatment, patients receiving esketamine had 43.2% more weeks in functional remission and 14.2% less overall loss of productivity at work than patients treated with quetiapine XR [22].

Improvements in social functioning and interpersonal relationships seem particularly important from a clinical perspective. Depression, especially treatment-resistant depression, are associated with limited social contacts, deterioration of family relationships, decreased professional activity, and loss of previously performed life roles. Consequently, improvement in the social domain is an important indicator of functional recovery, which is not always fully reflected in changes in MADRS or HDRS scores. Observations from the ESCAPE-TRD study, which demonstrated greater improvement in social functioning and other aspects of functioning in patients treated with esketamine, support this understanding [21]. However, caution should be exercised in directly translating improved quality of life into professional activity. This study did not directly assess absenteeism, attendance at work despite illness, or return to work, so the relationship between improved HRQoL and regained professional activity should be further analyzed.

Differences between groups should be interpreted not only in terms of statistical significance but also in terms of their clinical significance. In treatment-resistant depression, improved quality of life may mean not only reduced symptom severity but also increased ability to engage in daily activities, improved social relationships, greater independence, and a more favorable assessment of one’s health status. Therefore, quality of life measurements are an important complement to traditional symptom assessment and can provide information more directly related to patient-relevant treatment goals. The use of two complementary tools in this study further strengthens this interpretation: the WHOQOL-BREF captures the broad context of functioning and well-being, while the SF-36 allows for a more detailed assessment of health-related aspects of quality of life [16,17]. There is no single universal minimal clinically important difference (MCID) threshold for WHOQOL-BREF in TRD. In a study of patients with treatment-resistant depression, a practical 10-point threshold in WHOQOL-BREF domains (0–100 scale) was used as an indicative clinically meaningful change [23]. For SF-36 subscales, differences of approximately 3–5 points are often considered potentially clinically meaningful; however, the threshold depends on the population and domain, and its empirical basis is limited [24]. These thresholds were not prespecified in the present study; therefore, interpretation of clinical significance should be regarded as exploratory.

From a clinical perspective, the results support the inclusion of quality of life as one of the elements of monitoring treatment response in treatment-resistant depression. A reduction in depression scores alone does not always reflect the degree of recovery in daily life. Assessment of areas such as physical functioning, social relationships, mental health, vitality, and the ability to perform life roles may provide additional information needed to assess the actual effect of therapy. In this context, the WHOQOL-BREF and SF-36 may be useful tools in both clinical trials and clinical practice, provided their results are interpreted appropriately and the time constraints associated with their use are taken into account [16,17].

A strength of this study is the use of two complementary quality of life assessment tools. The WHOQOL-BREF encompasses a broad, multidimensional context of patient functioning, while the SF-36 allows for a detailed assessment of health-related aspects of quality of life. The simultaneous use of the MADRS and HDRS also enabled the comparison of changes in quality of life with changes in the severity of depressive symptoms. Another significant value of this study is its real-world nature and focus on the population of patients with treatment-resistant depression, which in clinical practice is often characterized by significant symptomatic and functional burden. Observational data can therefore complement the results obtained in controlled clinical trials, providing information on the effects of treatment in conditions closer to everyday practice.

This study has several limitations. First, the observational, nonrandomized, and unblinded design limits causal inference. Treatment selection reflected routine clinical decisions, and the study did not capture a standardized between-group measure quantifying the degree of SSRI dose optimization; the comparison therefore reflects two real-world treatment strategies rather than the isolated effect of esketamine. Confounding by indication and selection bias cannot be excluded, and unmeasured factors including prior treatment burden, episode chronicity, concomitant care, and psychosocial context may have influenced outcomes. Second, the sample was small (60 participants, 30 per group) and below the approximate a priori target for detecting a moderate between-group effect with 80% power. Third, cumulative esketamine exposure, short treatment interruptions, dose modifications, and longitudinal oral-antidepressant adherence were not captured as standardized exposure variables, limiting reconstruction of the full 12-month treatment course. Fourth, the large absolute quality-of-life changes observed in both groups may partly reflect regression to the mean, natural symptom fluctuation, expectancy effects, intensity of clinical contact, or concomitant treatment changes, and scores from different HRQoL instruments are not directly comparable. Fifth, missing data and loss to follow-up may introduce attrition bias, although 12-month retention was high in both groups. Sixth, dissociation was not assessed with a dedicated standardized scale and cognitive function was not a prespecified endpoint, preventing quantitative comparison of these safety domains with pivotal esketamine trials. Finally, multiple comparisons increase the risk of type I error; Holm correction was therefore applied to secondary WHOQOL-BREF and SF-36 comparisons. The findings should be viewed as comparative real-world evidence that complements, rather than replaces, randomized clinical-trial data. Future observational studies should prospectively record treatment exposure and adherence and should apply methods to reduce measured confounding, such as propensity-score matching or weighting, inverse-probability-of-treatment weighting, and adjusted longitudinal mixed-effects models. Sensitivity analyses and, where organizationally feasible, blinded outcome assessment are also warranted to reduce the risk of selection bias and residual confounding.

The study findings indicate that selection of a therapeutic strategy in treatment-resistant depression should consider not only changes in depressive symptom severity but also the patient’s functional goals and quality of life. A practical approach may include assessment with WHOQOL-BREF or SF-36 before initiation of a treatment strategy, repeat measurement after key treatment stages, and periodic reassessment during maintenance treatment, in parallel with MADRS/HDRS evaluation. For esketamine, implementation requires appropriate patient qualification, supervised administration, monitoring of blood pressure and adverse events, post-dose observation, trained staff, appointment availability, and consideration of the burden of frequent visits on the patient [15]. Discussions with patients should address not only symptom severity but also whether treatment enables return to activities and life roles that the patient considers important.

## 4. Material and Methods

### 4.1. Study Design

The study was a prospective, observational, comparative cohort study conducted in real-world clinical practice at two specialized psychiatric centers in Poland. Treatment exposure was not assigned by the investigators; patients were classified according to the strategy selected in routine clinical practice by the treating physician on the basis of clinical assessment, prior treatment history, clinical severity, medical indications, tolerability, comorbidities, concomitant treatment, patient preference, and treatment availability. Antidepressant pharmacotherapy remained under the responsibility of the treating physician. The study did not collect a standardized, between-group comparable measure of the degree of SSRI dose optimization. In the ESK group, esketamine was added to ongoing SSRI-based treatment, whereas the comparison group received SSRI-based treatment without esketamine. Because these factors could influence both treatment selection and outcomes, confounding by indication and selection bias cannot be excluded. The study was designed to compare outcomes associated with treatment strategies used in routine practice rather than to estimate an isolated causal effect of esketamine.

This study was reported in accordance with the STROBE guidelines [19]. A completed STROBE checklist is provided as Supplementary Material (Supplementary Table S1). Particular emphasis was placed on transparency of patient eligibility criteria, the definition of treatment exposure, data-collection procedures, follow-up time points, and limitations arising from the observational design.

### 4.2. Study Participants

Sixty adults with treatment-resistant depression were included in the analysis. The ESK group included 30 patients treated with intranasal esketamine as add-on therapy to ongoing SSRI-based antidepressant treatment, and the comparison group included 30 patients receiving SSRI-based treatment without esketamine. For this study, treatment-resistant depression was operationally defined as a current major depressive episode with insufficient clinical response despite at least two consecutive adequate antidepressant treatment trials during the current episode. An adequate trial required treatment at a therapeutic dose for an appropriate duration, generally at least 6 weeks per trial, with adherence documented in the medical record. TRD status was verified from available medical records and treatment history, including antidepressant agent, dose, duration, documented adherence, and clinical response to prior treatment.

Inclusion criteria were age 18–65 years, clinically confirmed major depressive disorder, fulfillment of the operational TRD definition, eligibility for one of the analyzed treatment strategies, ability to participate in scheduled follow-up, and availability of clinical and quality-of-life data. Exclusion criteria included bipolar disorder, psychotic symptoms, acute suicidal risk requiring urgent intervention, active substance-use disorder, clinically relevant somatic contraindications to the analyzed treatment, pregnancy or breastfeeding, conditions preventing reliable completion of psychometric questionnaires, substantial missing source documentation, and inability or unwillingness to comply with study procedures.

Sample-size calculation: An approximate a priori calculation for two independent groups assumed a moderate between-group effect (Cohen’s d = 0.5), two-sided alpha of 0.05, and 80% statistical power, indicating a target of approximately 64 participants per group. Owing to real-world recruitment feasibility, the final cohort included 30 participants per group (60 total). The study therefore had limited power to detect small effects and was not designed for extensive subgroup analyses; this should be considered when interpreting the findings.

#### 4.2.1. Demographic Characteristics of Individual Research Groups

The mean age of patients in the ESK group was 42.1 ± 10.5 years and in the SSRI group was 43.3 ± 11.2 years. Women comprised 60% of the ESK group and 63% of the SSRI group. There were no significant differences between the groups in terms of gender, education, or occupational status, supporting the comparability of the groups at baseline. Data are presented in Table 5.

#### 4.2.2. Clinical Characteristics at the Beginning of the Observation Period of the Individual Study Groups

At baseline, both groups had clinically significant increases in depressive symptoms. The mean MADRS score was 28.2 ± 4.5 in the ESK group and 27.8 ± 4.7 in the SSRI group. The mean HDRS score was 24.5 ± 3.8 and 24.1 ± 3.9, respectively. The duration of the current episode and the frequency of previous depressive episodes were comparable between the groups. Data are presented in Table 6.

### 4.3. Treatment with Esketamine and SSRIs

Patients in the ESK group received 84 mg of intranasal esketamine as add-on therapy to an SSRI antidepressant. The study protocol did not mandate a preference for a specific SSRI or a fixed dose-escalation algorithm. Selection of the oral SSRI and any dose modifications remained at the discretion of the treating physician and could be adjusted according to clinical response and tolerability; the detailed degree of dose optimization was not recorded as a standardized research variable comparable between groups. During the induction phase, esketamine was administered twice weekly for 4 weeks, then once weekly for the following 4 weeks. From week 9 onward, the frequency of administration was individualized by the treating physician according to clinical response and tolerability within routine clinical practice.

The patient self-administered the 84 mg dose under the direct supervision of medical personnel, using three disposable dispensers containing 28 mg of esketamine each. Each dispenser allowed for the administration of two sprays—one into each nostril—with a 5-min break between dispensers.

Esketamine was administered under direct medical supervision. Safety monitoring included clinical observation after each dose, blood-pressure measurement before and after administration, and documentation of adverse events during treatment. Adverse events were recorded according to type, severity, duration, need for intervention, and effect on treatment continuation. Particular attention was paid to transient dizziness or disorientation, nausea or vomiting, somnolence or fatigue, anxiety or inner restlessness, headache, and transient blood-pressure increases. Dissociative symptoms were not assessed using a dedicated standardized scale, and cognitive function was not a prespecified endpoint; consequently, dissociation rates and cognitive changes were not reported as quantitative study outcomes.

The comparison group received SSRI-based treatment without esketamine, managed by the treating physician according to routine clinical practice. The degree of SSRI dose modification or optimization was not standardized as a comparative research variable between groups.

### 4.4. Assessment Tools

The primary endpoint was health-related quality of life. WHOQOL-BREF and SF-36 were used as complementary patient-reported outcome instruments. WHOQOL-BREF assesses four domains: physical health, psychological health, social relationships, and environment. SF-36 was analyzed at the level of the standard subscales, including physical functioning (PF), role limitations due to physical health (RP), mental health/emotional well-being (MH), social functioning (SF), and bodily pain (BP) [16,17]. The same instrument versions, instructions, scoring rules, and prespecified time points were used in both groups. WHOQOL-BREF results were interpreted primarily at the domain level. Values transformed to a 0–100 scale and descriptive summary indicators were not treated as standardized effect sizes directly comparable with results from other quality-of-life instruments. PCS and MCS were not calculated for SF-36.

Secondarily, the severity of depressive symptoms was assessed using the MADRS and HDRS. The MADRS is particularly sensitive to changes during antidepressant treatment, whereas the HDRS remains a classic clinical tool used in depression research. In this article, the MADRS and HDRS scores are considered to provide interpretive context for changes in quality of life, rather than as the primary endpoint.

### 4.5. Time Points and Data Collection

Assessments were performed at baseline (T0), weeks 4, 8, and 12, and at the final 12-month follow-up (T_end). This schedule allowed assessment of both early treatment dynamics and longer-term maintenance of change. Data were obtained from clinical records, standardized psychometric assessments, and quality-of-life questionnaires. For each treatment group, the number of participants contributing data to the final assessment was explicitly recorded. Primary analyses were based on available observations at the respective time points; missing follow-up outcome values were not imputed in the primary analyses. Retention and the potential for attrition bias were considered when interpreting 12-month outcomes. Visit-level cumulative esketamine administrations, short treatment interruptions, dose modifications, and longitudinal changes in oral antidepressant dose/adherence were not predefined as standardized research variables and were therefore not retrospectively reconstructed.

### 4.6. Statistical Analysis

Quantitative variables were presented as mean ± standard deviation (SD), and variables with distributions deviating from normality as median and interquartile range (IQR). Categorical variables were presented as counts and percentages. Distributional assumptions for continuous variables were assessed before inferential statistical testing. Baseline between-group comparisons were performed using the independent-samples Student’s *t*-test for approximately normally distributed continuous variables and the Mann–Whitney U test when distributional assumptions were not met; categorical variables were compared using the chi-square test or Fisher’s exact test, as appropriate. Because outcomes were assessed repeatedly in the same participants at T0, weeks 4, 8, and 12, and T_end, longitudinal changes in MADRS, HDRS, and quality-of-life scores were analyzed using repeated-measures analysis of variance (RM-ANOVA), with time treated as the within-subject factor and treatment group (ESK vs. SSRI) as the between-subject factor. The group × time interaction was used to assess whether trajectories differed between treatment strategies. Greenhouse–Geisser correction was applied when the sphericity assumption was violated. For quality-of-life assessments, the absolute change from T0 to T_end and the between-group difference in change were reported. In addition to *p* values, descriptive between-group differences at T_end were presented with 95% confidence intervals and Cohen’s d effect sizes calculated from endpoint means and pooled standard deviations. In secondary analyses involving multiple WHOQOL-BREF domains and SF-36 subscales, multiplicity was addressed using the Holm procedure; both nominal and adjusted *p* values were reported. All tests were two-sided, and *p* < 0.05 was considered statistically significant. Statistical analyses were performed using TIBCO Statistica 13.3 (TIBCO Software Inc., Palo Alto, CA, USA).

### 4.7. Ethical Aspects

The study was conducted in accordance with the principles of the Declaration of Helsinki and applicable regulations on personal data protection. The study protocol received a positive opinion from the Bioethics Committee of the Medical University of Silesia in Katowice; decision no. BNW/NWN/0052/KB/104/25 of 25 March 2025. All participants provided written informed consent to participate in the study.

## 5. Conclusions

In this observational cohort of patients with treatment-resistant depression, a treatment strategy including intranasal esketamine as add-on therapy to SSRI-based treatment was associated with greater improvement in several quality-of-life measures than an SSRI-based strategy without esketamine. The observed differences involved WHOQOL-BREF domains and selected standard SF-36 subscales. Because treatment exposure was not assigned by the investigators, treatment modifications were not fully standardized as comparable exposure variables, and residual confounding, regression to the mean, and other limitations of the observational design cannot be excluded, the findings should not be interpreted as evidence of an isolated causal effect of esketamine. Confirmation is needed in larger prospective multicenter studies with prespecified longitudinal models, detailed treatment-exposure reporting, and comprehensive safety assessment.

## Figures and Tables

**Table 1:** Mean MADRS and HDRS scores during follow-up.

Time Point	MADRS ESK	MADRS SSRI	HDRS ESK	HDRS SSRI
Baseline (T0)	28.2 ± 4.5	27.8 ± 4.7	24.5 ± 3.8	24.1 ± 3.9
Week 4	21.4 ± 5.2	24.0 ± 4.9	18.3 ± 4.0	21.1 ± 4.2
Week 8	16.8 ± 5.0	21.5 ± 5.1	13.9 ± 3.7	18.4 ± 4.6
Week 12	13.5 ± 4.7	19.0 ± 4.9	10.7 ± 3.5	16.2 ± 4.3
Month 12 (T_end)	10.2 ± 3.8	16.5 ± 4.7	8.1 ± 3.2	13.7 ± 4.0

Values are presented as M ± SD.

**Table 2:** Descriptive WHOQOL-BREF summary indicator in the ESK and SSRI groups.

Indicator	ESK T0	ESK T_end	Δ ESK	SSRI T0	SSRI T_end	Δ SSRI	*p*	ESK–SSRI Difference at T_end (95% CI); Cohen’s d
WHOQOL-BREF, descriptive summary indicator	52.1 ± 6.3	75.4 ± 5.7	+23.3	51.7 ± 6.0	67.2 ± 5.9	+15.5	0.01	8.2 (5.2–11.2); d = 1.41

T_end—final assessment after 12 months; Δ—absolute change from T0 to T_end. The last column presents the descriptive between-group difference at T_end (ESK minus SSRI), its 95% confidence interval, and Cohen’s d calculated from endpoint means and pooled standard deviations. The *p* value refers to the between-group comparison of T0-to-T_end change using an independent-samples Student’s *t*-test or the Mann–Whitney U test, depending on data distribution. Longitudinal change was additionally assessed using RM-ANOVA as described in Section 4.6. The summary indicator is descriptive and does not replace interpretation of the official WHOQOL-BREF domains.

**Table 3:** Dynamics of WHOQOL-BREF domains.

Domain	ESK T0	ESK T_end	Δ ESK	SSRI T0	SSRI T_end	Δ SSRI	*p*	ESK–SSRI Difference at T_end (95% CI); Cohen’s d
Physical health	51.0 ± 7.1	76.5 ± 6.0	+25.5	50.5 ± 6.8	68.2 ± 6.1	+17.7	0.008 (0.032)	8.3 (5.2–11.4); d = 1.37
Psychological health	50.8 ± 6.5	74.2 ± 5.5	+23.4	51.2 ± 6.2	66.5 ± 5.8	+15.3	0.012 (0.032)	7.7 (4.8–10.6); d = 1.36
Social relationships	53.5 ± 6.0	77.0 ± 5.3	+23.5	52.8 ± 5.8	66.8 ± 6.0	+14.0	0.010 (0.032)	10.2 (7.3–13.1); d = 1.80
Environment	53.4 ± 5.9	75.2 ± 5.1	+21.8	53.1 ± 5.7	66.5 ± 5.5	+13.4	0.015 (0.032)	8.7 (6.0–11.4); d = 1.64

Values are presented as M ± SD unless otherwise indicated. In the *p* column, nominal *p* values are shown before Holm-adjusted values (in parentheses) for the four WHOQOL-BREF domain comparisons. The last column shows the descriptive between-group difference at T_end with its 95% confidence interval and Cohen’s d, calculated from endpoint means and pooled standard deviations. The *p* values refer to between-group comparisons of T0-to-T_end change using an independent-samples Student’s *t*-test or the Mann–Whitney U test, depending on data distribution. Longitudinal change was additionally assessed using RM-ANOVA as described in Section 4.6.

**Table 4:** Dynamics of selected SF-36 subscales.

Subscale	ESK T0	ESK T_end	Δ ESK	SSRI T0	SSRI T_end	Δ SSRI	*p*	ESK–SSRI Difference at T_end (95% CI); Cohen’s d
Physical functioning (PF)	45.2 ± 6.8	73.5 ± 5.9	+28.3	46.1 ± 7.0	65.2 ± 6.3	+19.1	0.007 (0.035)	8.3 (5.1–11.5); d = 1.36
Role limitations due to physical health (RP)	46.8 ± 7.2	71.8 ± 6.2	+25.0	45.5 ± 6.9	62.5 ± 6.7	+17.0	0.010 (0.040)	9.3 (6.0–12.6); d = 1.44
Mental health/emotional well-being (MH)	47.5 ± 6.5	72.0 ± 5.8	+24.5	48.0 ± 6.3	64.0 ± 6.2	+16.0	0.012 (0.040)	8.0 (4.9–11.1); d = 1.33
Social functioning (SF)	50.3 ± 6.2	74.5 ± 5.5	+24.2	49.5 ± 5.8	65.8 ± 5.7	+16.3	0.011 (0.040)	8.7 (5.8–11.6); d = 1.55
Bodily pain (BP)	50.5 ± 6.0	73.2 ± 5.4	+22.7	51.0 ± 5.7	65.0 ± 5.5	+14.0	0.013 (0.040)	8.2 (5.4–11.0); d = 1.50

The selected subscales correspond to areas particularly relevant to the functioning of patients with treatment-resistant depression. In the *p* column, nominal *p* values are shown before Holm-adjusted values (in parentheses) for the five SF-36 subscale comparisons. The last column shows the descriptive between-group difference at T_end with its 95% confidence interval and Cohen’s d, calculated from endpoint means and pooled standard deviations. The *p* values refer to between-group comparisons of T0-to-T_end change using an independent-samples Student’s *t*-test or the Mann–Whitney U test, depending on data distribution. Longitudinal change was additionally assessed using RM-ANOVA as described in Section 4.6.

**Table 5:** Demographic characteristics of patients at the beginning of the follow-up.

Indicator	ESK (*n* = 30)	SSRI (*n* = 30)	*p*
Age, years, M ± SD	42.1 ± 10.5	43.3 ± 11.2	0.67
Women, %	60	63	0.78
Men, %	40	37	0.78
Higher education, %	50	47	0.82
Secondary education, %	50	53	0.82
Employed, %	66	63	0.77

M ± SD—mean ± standard deviation; ESK—esketamine; SSRI—selective serotonin reuptake inhibitors.

**Table 6:** Clinical characteristics of patients at the beginning of the follow-up.

Indicator	ESK (*n* = 30)	SSRI (*n* = 30)	*p*
Duration of current episode, months, median (IQR)	9.5 [6,7,8,9,10,11,12,13,14]	10.2 [5,6,7,8,9,10,11,12,13,14,15]	0.68
MADRS, M ± SD	28.2 ± 4.5	27.8 ± 4.7	0.74
HDRS, M ± SD	24.5 ± 3.8	24.1 ± 3.9	0.71
>2 previous depressive episodes, %	40	43	0.79
Comorbid anxiety, %	33	30	0.78

MADRS—Montgomery–Åsberg Depression Rating Scale; HDRS—Hamilton Depression Rating Scale; IQR—interquartile range.

## Data Availability

The original contributions presented in this study are included in the article/Supplementary Materials. Further inquiries can be directed to the corresponding author.

## Supplementary Materials

The following supporting information can be downloaded at: https://www.mdpi.com/article/10.3390/ph19101644/s1, Supplementary Table S1. STROBE checklist for the observational cohort study.
