# Supplementary material for: Quality of Life in Patients with Treatment-Resistant Depression Treated with Intranasal Esketamine as Add-On Therapy to an SSRI—A Prospective, Comparative Study in Real-World Clinical Practice
Source: Pharmaceuticals (Basel). 2026 Oct 19;19(10):1644. doi: 10.3390/ph19101644 (PMC13708465; doi:10.3390/ph19101644)
Supplement: Supplementary file 1 [file pharmaceuticals-19-01644-s001.zip › pharmaceuticals-4592973-supplementary.pdf]

Supplementary Table S1. STROBE checklist for the observational cohort study

Manuscript: Quality of Life in Patients with Treatment-Resistant Depression Treated with Intranasal Esketamine as Add-On Therapy to an SSRI—A Prospective, Comparative Study in Real-World Clinical Practice

Completed against the revised manuscript supplied for resubmission to Pharmaceuticals.

**Item**	**STROBE topic**	**Where addressed in revised manuscript**	**Status/note**
1	Title and abstract	Title; Abstract	Design explicitly identified as prospective observational comparative cohort.
2	Background/rationale	Introduction, paragraphs 1–6	Clinical rationale and need for real-world HRQoL data described.
3	Objectives	End of Introduction	Primary and secondary objectives and hypothesis explicitly stated.
4	Study design	Section 4.1	Observational cohort design stated early.
5	Setting	Section 4.1; Section 4.5	Two specialist psychiatric centers in Poland; follow-up schedule stated.
6	Participants	Section 4.2	Eligibility, treatment groups, and clinical selection described.
7	Variables	Sections 4.3–4.5	Treatment exposure, HRQoL outcomes, MADRS/HDRS, time points described.
8	Data sources/measurement	Sections 4.4–4.5	WHOQOL-BREF, SF-36, MADRS, HDRS and data sources described.
9	Bias	Sections 4.1 and 2.6; Discussion – limitations	Confounding by indication, selection bias, attrition, regression to the mean, unblinded assessment and other potential biases are explicitly discussed.
10	Study size	Section 4.2	Approximate sample-size calculation and feasibility-limited recruitment reported.
11	Quantitative variables	Section 4.6	Continuous/categorical variable handling and summary statistics specified.
12a	Statistical methods – main	Section 4.6	t-test/Mann–Whitney, chi-square/Fisher, repeated-measures ANOVA described.
12b	Statistical methods – confounding	Section 4.1; Discussion—limitations	No multivariable or propensity-based confounding adjustment was performed; confounding by indication and selection bias are explicitly acknowledged.
12c	Subgroups/interactions	Section 4.6; limitations	Group × time interaction stated; extensive subgroup analyses not supported by sample size.
12d	Missing data	Section 2.6; Section 4.5	Twelve-month retention and missing observations are reported; analyses used available observations and no imputation was performed because follow-up missingness was small.

Item	STROBE topic	Where addressed in revised manuscript	Status/note
12e	Sensitivity analyses	Discussion—limitations	No formal sensitivity analyses were performed; this limitation is acknowledged and sensitivity analyses are recommended for future studies.
13	Participants through study	Sections 2.5–2.7	Sixty participants were included (30 per group); 12-month data were available for 29/30 participants in the ESK group and 28/30 in the SSRI group, with missingness and retention described.
14	Descriptive data	Tables 5–6; Section 2.6	Baseline demographic and clinical characteristics are reported by group, together with retention and missing-data information.
15	Outcome data	Tables 1–4	Depressive symptoms and HRQoL outcomes reported by time/group.
16	Main results	Results; Tables 2–4	Absolute changes, between-group differences, 95% confidence intervals, Cohen's d values, and multiplicity-adjusted p values are reported where applicable; causal language is avoided.
17	Other analyses	Sections 2.2–2.4; Section 4.6	Longitudinal, domain-level and subscale analyses are described, including group × time interaction and multiplicity adjustment.
18	Key results	Beginning of Discussion	Principal findings summarized in relation to objectives.
19	Limitations	Discussion—limitations	Direction and implications of major biases/limitations discussed.
20	Interpretation	Discussion	Cautious interpretation considering design and external evidence.
21	Generalisability	Discussion/limitations	Real-world relevance discussed; sample size and setting limit generalisability.
